# Effects of doxorubicin‐induced cardiotoxicity on cardiac mitochondrial dynamics and mitochondrial function: Insights for future interventions

**DOI:** 10.1111/jcmm.15305

**Published:** 2020-04-26

**Authors:** Nichanan Osataphan, Arintaya Phrommintikul, Siriporn C. Chattipakorn, Nipon Chattipakorn

**Affiliations:** ^1^ Division of Cardiology Department of Internal Medicine Faculty of Medicine Chiang Mai University Chiang Mai Thailand; ^2^ Cardiac Electrophysiology Research and Training Center Faculty of Medicine Chiang Mai University Chiang Mai Thailand; ^3^ Center of Excellence in Cardiac Electrophysiology Research Chiang Mai University Chiang Mai Thailand; ^4^ Cardiac Electrophysiology Unit Department of Physiology Faculty of Medicine Chiang Mai University Chiang Mai Thailand

**Keywords:** cardiotoxicity, doxorubicin, mitochondrial dynamics, mitochondrial function

## Abstract

Anthracyclines is an effective chemotherapeutic treatment used for many types of cancer. However, high cumulative dosage of anthracyclines leads to cardiac toxicity and heart failure. Dysregulation of mitochondrial dynamics and function are major pathways driving this toxicity. Several pharmacological and non‐pharmacological interventions aiming to attenuate cardiac toxicity by targeting mitochondrial dynamics and function have shown beneficial effects in cell and animal models. However, in clinical practice, there is currently no standard therapy for the prevention of anthracycline‐induced cardiotoxicity. This review summarizes current reports on the impact of anthracyclines on cardiac mitochondrial dynamics and mitochondrial function and potential interventions targeting these pathways. The roles of mitochondrial dynamics and mitochondrial function in the development of anthracycline‐induced cardiotoxicity should provide insights in devising novel strategies to attenuate the cardiac toxicity induced by anthracyclines.

**Main topics**

- Mitochondria are one of the main regulator in the development of doxorubicin‐induced cardiotoxicity.
- Doxorubicin inhibits mitochondrial fusion, promotes mitochondrial fission and impairs mitochondrial function which subsequently leads to heart failure.
- Targeting mitochondria by modulation of mitochondrial dynamics and mitochondrial function could be a promising strategy to attenuate the cardiotoxicity from doxorubicin

## 1 INTRODUCTION

Anthracyclines are potent chemotherapeutic agents for treatment of multiple solid and haematologic malignancies. Despite their high efficacy in cancer treatment, anthracyclines can induce cardiotoxicity in both acute and chronic forms. Acute cardiotoxicity has been described as arrhythmia and transient left ventricular dysfunction, but these are rare as compared to chronic cardiotoxicity.^1^, ^2^ High cumulative doses of anthracyclines can lead to left ventricular systolic dysfunction and heart failure. The highest incidence was observed in doxorubicin therapy in which a sharp rise in left ventricular systolic dysfunction occurred when the cumulative dose of doxorubicin reached 400 mg/m^2^.^3^ The reported incidence of heart failure is 5%, 26% and 48% in patients receiving 400, 550 and 700 mg/m^2^ of doxorubicin, respectively.^4^ From a recent prospective cohort study,^5^ the incidence of left ventricular systolic dysfunction was found to be 9% and the majority of the cases (98%) occurring within the first year of treatment. It has been shown that the risk of heart failure could be cumulative for up to 30 years after exposure to anthracyclines.^6^ Currently, there is no standard effective therapy for the prevention of anthracycline‐induced cardiotoxicity.

There are several proposed mechanisms for doxorubicin‐induced cardiotoxicity. Doxorubicin primarily targets topoisomerase IIβ (Top2β) and induces DNA double‐strand breaks.^7^ It also disrupts cardiac pro‐survival pathway, the neuregulin/ErbB signalling which results in mitochondrial dysfunction and apoptosis.^8^, ^9^ Moreover, the most pronounce mechanism responsible for doxorubicin‐induced cardiotoxicity is the formation of reactive oxygen species (ROS), leading to oxidative stress.^10^ The generation of ROS occurs via multiple pathways.^10^ Mitochondria are subcellular organelle, which are the main source and target of ROS.^11^ Several studies indicated that doxorubicin‐induced mitochondrial dysfunction, increased production of ROS which subsequently led to the development of cardiac muscle dysfunction and heart failure.^12^, ^13^ Mitochondria occupy about 40% of each cardiomyocyte volume, and the majority of the energy produced in the cardiomyocyte is from mitochondrial respiration.^14^, ^15^ Doxorubicin can target to the mitochondria mainly through ROS production.^16^ ROS induces mitochondrial DNA damage and decreases mitochondrial transmembrane potential.^12^, ^17^ In addition, doxorubicin also directly interferes with mitochondrial function by inhibiting electron transport chain proteins expression and promotes mPTP opening.^12^, ^18^ Inhibition of mitochondrial function by doxorubicin contributes to cardiac energy starvation and cell death.^12^ All of these data suggest that mitochondria are one of the main regulators in the development of doxorubicin‐induced cardiotoxicity.

Mitochondria are dynamic organelles whose primary function is ATP production.^19^ Mitochondria protect cells against cellular stress by several processes. Mitochondrial transcription factors such as peroxisome proliferator‐activated receptor‐γ coactivator 1α (PGC‐1α) and mitochondrial transcription factor A (TFAM) mediate an increase in mitochondrial number in response to cellular damage.^20^, ^21^ This process is called mitochondrial biogenesis which could regulate mitochondrial dynamics. Mitochondrial dynamics consists of mitochondrial fusion and fission. Maintaining balance of mitochondrial dynamics is a key for achieving normal mitochondrial function by controlling several aspects including mitochondrial respiration, mitochondrial metabolism and ROS production.^22^ Mitochondrial dynamics are regulated by guanosine triphosphatases (GTPases) in the dynamin family. Fusion is mediated by mitofusin‐1 (MFN1) and mitofusin‐2 (MFN2) proteins at the outer mitochondrial membrane and optic atrophy 1 (OPA1) at the inner mitochondrial membrane.^19^ Mitofusins initiate fusion by the process of mitochondrial tethering of two adjacent mitochondria and create homodimeric or heterodimeric complexes of MFN1 and MFN2 at the outer mitochondrial membrane.^23^ After fusion of the outer mitochondrial membrane, OPA1 mediates the inner mitochondrial membrane fusion resulting in a more interconnected mitochondrial network. With regards to mitochondrial fission, DRP1 is recruited from the cytosol and binds to DRP1 receptor proteins at the outer mitochondrial membrane including mitochondrial fission protein1 (MTFP1) and mitochondrial fission factor (MFF).^24^ Fission leads to mitochondrial fragmentation and enhances the generation of ROS. There is growing evidence that doxorubicin disrupts mitochondrial dynamics and mitochondrial function.^14^, ^25^

In this review, the effects of doxorubicin on mitochondrial dynamics and mitochondrial function have been comprehensively summarized. Consistent and controversial reports from in vitro and in vivo models have been presented. The proposed pharmacological interventions to ameliorate doxorubicin‐induced cardiotoxicity have been presented and discussed.

## 2 DOXORUBICIN‐INDUCED CARDIOTOXICITY AND CARDIAC MITOCHONDRIAL DYNAMICS: EVIDENCE FROM IN VITRO AND IN VIVO STUDIES

Interference with mitochondrial dynamics is associated with the development of cardiovascular and metabolic diseases including ischaemia‐reperfusion injury, metabolic syndrome and also in doxorubicin‐induced cardiotoxicity.^26^, ^27^, ^28^, ^29^ Doxorubicin increased mitochondrial fragmentation followed by ROS generation and apoptosis.^14^, ^25^, ^30^ These effects of doxorubicin could be explained by an imbalance of mitochondrial dynamics. In vitro studies in neonatal rat cardiomyocytes (NRCMs) treated with doxorubicin dosage between 0.86 and 1.72 µmol/L for 1‐24 hours showed a significant decrease in mitochondrial fusion proteins, MFN1, MFN2, OPA1 and increase in phosphorylation of DRP1 at serine 616.^14^, ^30^ Using H9c2 cell and postnatal rat cardiomyocytes also showed consistent result that phosphorylation of DRP1 at serine 616 was increased.^29^, ^31^ These findings indicated that doxorubicin inhibited mitochondrial fusion and promoted mitochondrial fission. There are some conflicting results regarding mitochondrial fusion. Rats treated with doxorubicin dosage 2 mg/kg/wk for 7 weeks showed suppression of MFN1, MFN2 and OPA1 proteins expression.^13^, ^32^ Another in vivo study in mice treated with single dose of doxorubicin (10 mg/kg) showed that doxorubicin increased RNA expression of *Mfn2* and *Opa1*.^13^ Differences between dosage and analytical methods may explain the discrepancy in results found concerning the mitochondrial fusion process.

The precise mechanism of how doxorubicin regulates mitochondrial dynamics proteins is unclear. It has been shown that DRP1 translocation is controlled by MTFP1.^28^ Transfection of HL‐1 cardiac cell with *Mtfp1*‐shRNA inhibited mitochondrial fission and apoptosis.^28^ Interestingly, recent evidence showed that sirtuin‐3 (SIRT3) could regulate mitochondrial dynamics. SIRT3 is a protein that possess a deacetylase activity and is responsible for deacetylating of several mitochondrial proteins including OPA1.^25^ Acetylation of OPA1 represses OPA1 function. Transfection of the NRCMs with Ad‐*Sirt3* showed a decrease in OPA1 acetylation and apoptosis.^25^ This finding suggested that SIRT3 may have a protective role in a doxorubicin‐induced cardiotoxicity model. Several studies have proposed the role of microRNA in the regulation of mitochondrial dynamics. Cardiomyocytes isolated from rat hearts showed that MFN1 was negatively controlled by microRNA‐140.^30^ Another in vitro study using NRCMs indicated that microRNA‐532‐3p promoted mitochondrial fission by suppressing the expression of apoptosis repressor with caspase recruitment domain (ARC).^33^ Further studies are needed to investigate the role of different microRNA in this model. Overall, evidence from in vitro and in vivo models indicated that doxorubicin caused an imbalance in mitochondrial dynamics by inhibiting mitochondrial fusion and promoting mitochondrial fission. The findings from these reports are comprehensively summarized in Table 1.

**Table 1:** Effects of Doxorubicin on mitochondrial dynamics: reports from in vitro and in vivo studies

Study model	Methods (Drug/Dose/Route/Duration)	Major Findings			Interpretation	Ref
		Mitochondrial fusion	Mitochondrial fission	ROS/Apoptosis		
**in vitro reports**						
Isolated 1‐ to 4‐d‐old neonatal Sprague Dawley cardiomyocytes	• Dox/0.86‐1.72 µmol/L/8‐24 h Transfected with *Mfn2* • Dox/1.72 µmol/L/4‐24 h	↓MFN2 ↑MFN2	↑Mitochondrial fragmentation ↓Mitochondrial fragmentation	↑ROS ↑Caspase‐3 activity ↑TUNEL staining ↓ROS ↓Caspase3 activity ↓TUNEL staining	Dox decreased MFN2 expression which promoted mitochondrial fission, ROS production and apoptosis	14
1‐d‐old neonatal Wistar rats cardiomyocytes	• Dox/1.0 µmol/L/1‐15 h Transfected with anti‐miR‐140 • Dox/1.0 µmol/L/1‐12 h Transfected with *Mfn1* • Dox/1.0 µmol/L/5‐15 h	↓MFN1 ↓MFN2 ↓OPA1 ↑MFN1 ↔MFN2 ↔OPA1	↑Mitochondrial fragmentation ↑DRP1 ↔DRP1 ↓Mitochondrial fragmentation	↑TUNEL staining ↓TUNEL staining	MFN1 was negatively controlled by miR‐140 and could regulate mitochondrial fission and apoptosis	30
Neonatal rat cardiomyocytes	• Dox/0.1‐0.3 µmol/L/24 h Transfected with Ad‐*Sirt3* • Dox/0.1‐0.3 µmol/L/24 h	↑OPA1 acetylation ↓OPA1 acetylation		↑TUNEL‐positive cells ↓TUNEL‐positive cells	SIRT3 overexpression blocked Dox‐mediated cell death by reducing OPA1 acetylation	25
Postnatal rat cardiomyocytes	• Dox/10 µmol/L/18 h Transfected with *Bnip3*‐shRNA • Dox/10 µmol/L/18 h		↑pSer616 DRP1 ↑Mitochondrial fragmentation ↓Mitochondrial fragmentation		BNIP3 was a critical mediator of mitochondrial fragmentation induced by Dox	29
H9c2 cell	• Dox/5 µmol/L/24 h Treated with Mdivi‐1/1 µmol/L/30 min • Dox/5 µmol/L/24 h		↑pSer616DRP1/DRP1 ↓pSer616DRP1/DRP1	↑Annexin V ↑Cleaved caspase3 ↓Annexin V ↓Cleaved caspase3	Mdivi‐1 blunted the increase in mitochondrial fission caused by Dox treatment	31
HL‐1 cell	•Dox/1‐2 µmol/L/4‐24 h Transfected with *Mtfp1*‐shRNA • Dox/2 µmol/L/24 h Transfected with *Mtfp1* cDNA • Dox/0.3 µmol/L/24 h		↑MTFP1 ↑Mitochondria DRP1 ↓Cytosol DRP1 ↑Mitochondrial fission ↓Mitochondria DRP1 ↑Cytosol DRP1 ↓Mitochondrial fission ↑Mitochondrial fission	↑Cleaved caspase3 ↑Cleaved PARP1 ↑DNA fragmentation ↓TUNEL‐positive cells ↓DNA fragmentation ↑TUNEL‐positive cells ↑DNA fragmentation	MTFP1 was associated with DRP1activation and mediated the signal required for Dox‐induced mitochondrial fission and apoptosis	28
**in vivo reports**						
Male C57BL/6 mice	• Dox/10 mg/kg/ip/single dose (Follow‐up 1.5 wk)	↑*Mfn2* ↑*OPA1* (RNA level)	↑*Mtfp1* ↔*Drp1* ↑Mitochondrial fragmentation		Dox increased *Mfn2*, *Opa1* and mitochondrial fission	13
Male Balb/c mice 8 weeks old	•Dox/15 mg/kg/ip/ 3 times/wk/2 wk		↑pSer616DRP1	↑Cleaved caspase3	Dox increased phosphorylation of DRP1 and increased apoptosis	31
6‐week‐old male Sprague Dawley rat	•Dox/2 mg/kg/wk/ip/7 wk	↓MFN1 ↓MFN2 ↓OPA1	↑DRP1		Dox decreased mitochondrial fusion proteins and increased DRP1	32

Abbreviations: BNIP3, BCL2/adenovirus E1B 19 kD protein‐interacting protein 3; Dox, Doxorubicin; DRP1, Dynamin‐related protein1; MFN1, Mitofusin1; MFN2, Mitofusin2; MTFP1, Mitochondrial fission protein1; OPA1, Optic atrophy1; PARP, Poly (ADP‐ribose) polymerase; pSer616, Phosphorylation serine616; ROS, Reactive oxygen species; SIRT3, Sirtuin3; TUNEL, Terminal deoxynucleotidyl transferase‐mediated Dutp nick end‐labelling.

## 3 DOXORUBICIN‐INDUCED CARDIOTOXICITY AND CARDIAC MITOCHONDRIAL DYSFUNCTION: EVIDENCE FROM IN VITRO AND IN VIVO STUDIES

Molecular mechanisms associated with doxorubicin‐induced cardiotoxicity are multifactorial and complex.^34^ After several decades of investigation, doxorubicin‐induced cardiotoxicity is still a necessary focus in the field of cardio‐oncology research as the precise mechanism associated with its cardiotoxicity remains unclear. Increased oxidative stress has been shown as a key feature of doxorubicin‐induced heart failure.^35^, ^36^ Cardiomyocytes are known to be more susceptible to oxidative damage than other cells due to their lower capacity for antioxidant defence and their high density of mitochondria, which are the main source of ROS generation.^37^, ^38^ The chemical structure of doxorubicin is susceptible to redox cycling. As it is a quinone compound, doxorubicin is reduced by oxidoreductases within the cell including NADPH oxidase, xanthine oxidase and mitochondrial electron transport chain enzymes, especially complex I.^39^ Redox cycling leads to the formation of a semiquinone compound which interacts with oxygen to form the superoxide anion.^40^ The generation of ROS induces lipid peroxidation at the cellular membrane and has impact on other subcellular organelles including the nucleus and mitochondria.^16^ Excessive production of ROS causes oxidative damage to mitochondrial DNA (mtDNA),^17^, ^41^, ^42^ increased mitochondrial depolarization^12^, ^29^, ^36^, ^43^, ^44^, ^45^, ^46^, ^47^, ^48^, ^49^, ^50^, ^51^ and alteration in the morphology of mitochondria as indicated by mitochondrial swelling and fragmentation.^17^, ^25^, ^31^, ^41^, ^44^, ^45^, ^52^ The negative consequences of mitochondrial dysfunction result in cardiac contractile dysfunction indicated by both reduced left ventricular fractional shortening and ejection fraction.^13^, ^17^, ^31^, ^41^, ^44^, ^45^, ^52^, ^53^, ^54^, ^55^, ^56^, ^57^, ^58^, ^59^, ^60^, ^61^, ^62^ These could be the effect of ROS production and dysregulation of calcium channels or transporters, which are susceptible to redox cycling.^63^ Furthermore, doxorubicin interferes with mitochondrial respiration at electron transport chain (ETC) level by inhibiting complex I, II, IV proteins ^18^ and complex I activity.^12^, ^31^, ^43^, ^55^, ^64^, ^65^ These ETC complexes are necessary for the process of oxidative phosphorylation which is the major source of ATP production. Alteration in mitochondrial respiration was observed in doxorubicin models as shown by an increase in state 4, and a decrease in state 3 respiration and respiratory control ratio.^13^, ^54^, ^60^, ^65^ These findings indicated that doxorubicin inhibited mitochondrial respiration and led to mitochondrial bioenergetics failure. There is evidence to suggest that inhibition of oxidative phosphorylation is secondary to dysregulation in mitochondrial calcium homeostasis regulated by an opening of mitochondrial permeability transition pores (mPTP).^66^ Oxidative stress induces mPTP opening and increases permeability of the inner mitochondrial membrane, thus allowing passage of small molecules into the membrane, leading to matrix swelling, mitochondrial transmembrane potential disruption and release of calcium from the matrix.^13^, ^29^, ^32^, ^35^, ^44^, ^47^, ^50^ Recent evidence found that the opening of mPTP was induced by BCL2/adenovirus E1B 19 kD protein‐interacting protein 3 (BNIP3).^29^, ^44^ Knockdown of *Bnip3* in mice treated with doxorubicin (20 mg/kg single dose) inhibited mPTP opening and restored mitochondrial function.^44^ These findings suggested that BNIP3 was responsible for the cardiotoxic effects of doxorubicin and could be a molecular target for the attenuation of doxorubicin‐induced cardiotoxicity. Furthermore, several studies also investigated the effects of doxorubicin on mitochondrial function using human induced pluripotent stem cell‐derived cardiomyocytes (hiPSC‐CMs). Consistent with the results found in other cell types, doxorubicin decreased mitochondrial membrane potential, increased mitochondrial calcium loading and decreased cell viability in these hiPSC‐CMs.^67^, ^68^ Suppression of *Sirt3* in hiPSC‐CMs also increased the toxicity of doxorubicin ^69^ which suggested the protective role of SIRT3, and this finding is also consistent with previous reports using NRCM and H9C2 cells.^18^, ^25^ A summary of these reports is shown in Table 2.

**Table 2:** Effects of Doxorubicin on mitochondrial function: reports from in vitro and in vivo studies

Study model	Methods (Drug/Dose/Route/Duration)	Major Findings					Interpretation	Ref
		Heart function/Morphology	Oxidative phosphorylation	AutophagyMitophagy	ROS/Apoptosis	MMP/mPTPMitochondrial protein		
**in vitro reports**								
Neonatal rat cardiomyocyte	• Dox/0.1‐0.3 µmol/L/24 h **Transfected with Ad‐*Sirt3*** • Dox/0.1‐0.3 µmol/L/24 h	↑Fragmented Mt ↑Swollen Mt ↓Fragmented Mt ↓Swollen Mt					SIRT3 expression preserved mitochondrial morphology after Dox treatment	25
Postnatal rat cardiomyocyte	• Dox/10 µmol/L /18 h ***Bnip3*‐shRNA** • Dox/10 µmol/L/18 h				↑ROS ↑LDH ↑%Dead cells ↓%Dead cells	↓MMP ↑mPTP ↑Mitochondria BNIP3	Dox induced mitochondrial dysfunction and increased mitophagy activity through BNIP3 activation	29
Isolated postnatal rat cardiac myocytes from 1‐ to 2‐d‐old Sprague Dawley rats	• Dox/5 or 10 µmol/L/18 h **Ad‐*Bnip3*** • Dox/5 or 10 µmol/L/18 h ***Bnip3*‐shRNA** • Dox/5 or 10 µmol/L/18 h		↓OCR ↓RRC ↓COX1‐UCP3 ↓OCR ↓RRC ↑OCR ↑RRC		↑ROS ↑LDH ↑Cardiac troponin T ↓Cell viability ↓LDH ↓Cardiac troponin T ↑Cell viability	↓MMP ↑mPTP ↑Mitochondria BNIP3 ↑MMP ↓mPTP	Dox‐induced mitochondrial respiratory chain defect was linked to BNIP3 activation	44
Human right atrial trabeculae	• Dox/1 µmol/L/0‐90 min	↓Developed force ↓Maximal contraction velocity ↓Maximal relaxation velocity	↑State 2 ↓State 3 ↓RCR			↓MMP ↑mPTP	Dox induced mitochondrial permeability transition pore opening and contractile dysfunction	50
H9c2 cell	• Dox/0.5‐1 µmol/L/6‐24 h				↑ROS ↑Nuclear p53 ↑Bax ↑Cytosolic cytochrome c ↑Caspase3,9 activity ↓DNA synthesis	↓MMP	Dox induced mitochondrial dysfunction and activation of apoptotic pathway	51
H9c2 cell	• Dox/1 µmol/L/24 h				↑ROS ↑Bax ↑Cytosolic cytochrome c ↑Cleaved caspase3 ↓Cell viability	↓MMP ↓HO1 ↓ARE activity ↓Nuclear NRF2 ↔Cytosol Keap1	Dox induced mitochondrial dysfunction, decreased HO1 protein expression and nuclear NRF2 translocation	49
H9c2 cell	• Dox/1‐10 µmol/L/24 h		↓Complex I,II,IV protein		↓SOD2 ↑ROS	↓SIRT3 ↓SIRT1 ↑PGC1α acetylation	Dox inhibited SIRT3, SIRT1 expression, mitochondrial respiration and increased ROS production	18
Human adult ventricular cardiomyocyte (AC16)	• Dox/250 nmol/L/24 h			↑LC3‐II/LC3‐I ↑Beclin1 ↓p62 ↑PINK1 ↑Parkin	↓ROS ↓MMP ↓Cell viability	↓PGC‐1α ↓NRF1 ↓TFAM	Dox‐induced mitophagy and autophagosome formation which resulted in decreased mitochondrial biogenesis proteins expression and mitochondrial damage	74
H9c2 cell	• Dox/3 µmol/L/24 h			↑LC‐3I ↑LC‐3II ↔LC‐3II/LC‐3I ↑p62	↑ROS ↑Caspase activity ↓Cell viability		Dox reduced autophagic activity, increased ROS and decreased cell viability	77
Neonatal cardiomyocyte	• Dox/1 µmol/L/24 h • Dox/1 µmol/L/24 h + Medium APN/30 µg/mL/24 h **AMPK inhibitor 1 µmol**/L • Dox/1 µmol/L/24 h + High APN/100 µg/mL/24 h				↓Bcl2 ↑Bax ↑Cytosol cytochrome c ↑Cleaved caspase3 ↑TUNEL‐positive ↑Bcl2 ↓Bax ↓Cytosol cytochrome c ↓Cleaved caspase3 ↓TUNEL‐positive ↓Bcl2 ↑Bax ↑Cytosol cytochrome c ↑Cleaved caspase3 ↑TUNEL‐positive	↓p‐AMPKα ↑p‐AMPKα ↓p‐AMPKα	APN had cardioprotective effects against Dox‐induced cardiomyopathy, and these effects could be involved in the regulation of AMPK signalling pathway	56
Neonatal Sprague Dawley rats cardiomyocyte	• Dox/1 µmol/L/2‐24 h				↑p‐p53 ↑Bax ↓Bcl‐2 ↑Caspase3 ↑TUNEL‐positive cells	↓MMP ↑p‐AMPKα (2 h) ↑AMP/ATP ↑p‐ACC	Dox‐induced cardiomyocyte apoptosis by increased p‐AMPKα	59
H9c2 cell	• Dox/10 nmol/L/1‐72 h				↑ROS ↑LDH ↓Cell viability	↔p‐AMPK (72 h) ↔p‐ACC ↔PKA activity ↔p‐PDGFRβ	Dox induced ROS production but had no significant changes in the AMPK signalling pathway	83
Human pluripotent stem cell‐derived cardiomyocytes (hiPSC‐CMs)	• Dox/1 µmol/L/24 h **Ad‐ *Sirt3*** • Dox/1 µmol/L/24 h **Ad‐ *Sirt3* shRNA** • Dox/1 µmol/L/24 h				↑ROS ↓MMP ↑ROS ↓MMP ↑↑ROS ↓↓MMP	↓↓ATP ↑Apoptosis cell ↓ATP ↑Apoptosis cell ↓↓↓ATP ↑↑Apoptosis cell	SIRT3 is negatively correlated to Dox‐induced cardiotoxicity through the regulation of ATP, MMP, ROS level and apoptosis in hiPSC‐CMs	69
Human pluripotent stem cell‐derived cardiomyocytes (hiPSC‐CMs)	• Dox/3‐10 µmol/L/16‐24 h				↓MMP ↑DNA double‐strand breaks ↑Intracellular calcium	↑Apoptosis cell	Dox increased apoptotic cell death, ROS production, mitochondrial dysfunction and intracellular calcium in hiPSC‐CMs	68
Human pluripotent stem cell‐derived cardiomyocytes (hiPSC‐CMs)	**Acute model **(Evaluated immediately after Dox) • Dox/150 or 300 nmol/L/every 2 d/3 doses **Chronic model **(Evaluated at Day 14) • Dox/150 or 300 nmol/L/every 2 d/3 doses				↔MMP ↔Mitochondrial calcium ↓MMP ↑Mitochondrial calcium	↓Cell number ↓Cell number	Dox affected cell viability and caused mitochondrial disturbances in hiPSC‐CMs which was most profound in the chronic model	67
**in vivo reports**								
Male C57BL/6 mice	• Dox/10 mg/kg/ip/single dose (Follow‐up 1.5 wk)	↓LVFS ↑Mt density ↓Mt size ↓Mt elongated	↓RCR			↑mPTP ↑PGC1α ↑Mt DNA	Dox decreased myocardial contractile function, mitochondrial function and increased mPTP opening	13
8‐week‐old male Balb/c mice	• Dox/15 mg/kg/ip/3 times/wk/2 wk	↓LVEF ↑LVEDD ↑LVESD ↑Mt width ↔Mt length ↓Mt Length/width ratio	↓Complex I activity ↔Complex IV activity		↑Cleaved caspase3 ↑TUNEL staining		Dox decreased LV function, mitochondrial respiration and increased apoptosis	31
6‐week‐old male Sprague Dawley rats	• Dox/2 mg/kg/wk/ip/7 wk			↑Beclin1 ↑Beclin1/Bcl2 ↑LC3‐II ↑PINK1 ↑p62	↑Caspase3,9 ↑Bax/Bcl2 ratio	↑mPTP	Dox increased autophagic activity and apoptosis	32
Male Sprague Dawley rats	• Dox/4 mg/kg/dose/ip/every 5 d/3 doses (Total 12 mg/kg) **Ad‐ SESN2** • Dox/4 mg/kg/dose/ip/every 5 d/3 doses (Total 12 mg/kg)	↓LVEF ↑LVEF		↑PINK1 ↓Parkin ↓p62 ↓LC3II ↓PINK1 ↑Parkin ↑p62 ↑LC3II		↓Sestrins2 ↑Sestrins2	Overexpression of SESN2 protected against Dox‐induced cardiotoxicity by alleviating Dox‐induced inhibition of Parkin‐mediated mitophagy	76
Mice aged 8‐10 wk	• Dox/20 mg/kg/ip/single dose ***Bnip3 ‐/‐* mice** • Dox/20 mg/kg/ip/single dose	↑Swollen Mt ↑Loss of Mt cristae ↑Mt vacuolization ↓LVFS Intact cristae ↓Mt vacuolization ↑LVFS	↓OCR ↓RRC ↑OCR ↑RRC		↑LDH ↓Survival ↓LDH ↑Survival	↑BNIP3	Dox activated BNIP3 and induced mitochondrial respiratory chain defects in mouse hearts	44
C57BL mice	• Dox/15 mg/kg/ip/single dose	↓LVEF ↓FS ↑Myocardial swelling and vacuolization			↑Lipid peroxidation ↑Protein carbonylation ↓GSH/GSSG ↑Cytosolic cytochrome c ↑TUNEL‐positive	↓PGC1α ↓NRF‐1 ↓MtDNA copy number	Dox induced cardiac contractile dysfunction and apoptosis through a decrease in mitochondrial biogenesis	41
8‐week‐old female C57BL6 mice	• Dox/8 mg/kg/wk/ip/4 wk		↓Complex I,II,IV protein		↓SOD2	↓SIRT3	Dox reduced SIRT3 expression, oxidative phosphorylation and SOD2 expression	18
LC3 transgenic mice inoculated with E0771 cells	• Dox/20 mg/kg/ip/split into 2 doses			↓LC‐3II/LC‐3I ↑p62	↑Cleaved caspase3 ↓Survival ↓Tumour growth		Dox reduced autophagic activity, decreased survival and tumour growth	77
C57BL/6 mice	**WT mice** • Dox/4 mg/kg/wk/6 wk **APN transgenic sense (APN‐SE)** • Dox/4 mg/kg/wk/6 wk **APN transgenic antisense (APN‐AS)** • Dox/4 mg/kg/wk/6 wk	↓LVFS ↑Myocardial fibrosis ↑LVFS ↓Myocardial fibrosis ↓↓LVFS ↑↑Myocardium fibrosis			↓Bcl2 ↑Bax ↑Caspase3 ↓Survival ↑Bcl2 ↓Bax ↓Caspase3 ↑Survival ↓↓Bcl2 ↑↑Bax ↑↑Caspase3 ↓↓Survival	↓p‐AMPKα ↑p‐AMPKα ↓↓p‐AMPKα	APN had cardioprotective effects in Dox‐induced cardiomyopathy and was involved in the AMPK signalling pathway	56
Male Sprague Dawley rats	• Dox/20 mg/kg/ip/single dose	↓LVEDV ↓Stroke volume ↓LVEF			↑p‐p53 ↓Bcl2 ↑Cleaved caspase3 ↑TUNEL‐positive ↓Survival	↑p‐AMPKα	Dox increased p‐AMPKα and apoptosis	59
Male Wistar Albino rats	• Dox/20 mg/kg/ip/divided 2 doses/day 2 and 4	↑Myocyte degeneration ↑Interrupted muscle fibre ↑Wide interstitial spaces			↓Bcl‐2 ↑Caspase3 ↑CK‐MB ↑LDH ↓GSH ↑TBA	↑TNF‐α	Dox increased myocardial damage, ROS generation and apoptosis	73
Male Wistar rats	• Dox/15 mg/kg/ip/single dose	↑Myocardial degeneration ↑Interstitial inflammation ↑Interstitial haemorrhage			↑LDH ↑CK‐MB ↑MDA ↓SOD ↑Caspase3		Dox induced myocardial damage, oxidative stress and apoptosis	107
Sprague Dawley rats	• Dox/3 mg/kg/EOD/ip/6 doses	↓Aortic flow ↓Cardiac output ↓Stroke volume ↓Myocardial thickness		↔Beclin‐1 ↓LC3B‐II ↑p62	↔LDH ↔CK‐MB ↑Troponin T ↔MDA	↔p‐AMPK	Dox impaired the autophagic process, increased oxidative damage and cardiac dysfunction	78

Abbreviations: ACC, Acetyl‐CoA carboxylase; APN, Adiponectin; ARE, Antioxidant‐responsive elements BNIP3, BCL2/adenovirus E1B 19 kD protein‐interacting protein 3; CAT, Catalase; COX1, cytochrome c oxidase subunit1; Dox, Doxorubicin; FHC, Ferritin heavy chain; GSH, Reduced glutathione; GSSG, Oxidized glutathione; GSTα, Glutathione S‐transferase‐α; HO1, Haem oxgenase1; Keap1, Kelch‐like ECH‐associated protein 1; LVEDD, Left ventricular end‐diastolic dimension; LVEDV, Left ventricular end‐diastolic volume; LVEF, Left ventricular ejection fraction; LVESD, Left ventricular end‐systolic dimension; LVFS, Left ventricular fractional shortening; MDA, Malondialdehyde; MMP, Mitochondrial membrane potential; mPTP, Mitochondrial permeability transition pore; Mt, Mitochondria; NRF, Nuclear respiratory factor; OCR, Oxygen consumption rate; P, phosphorylation; PDGFRβ, Platelet‐derived growth factor receptor β; PKA, Protein kinase A; RCR, Respiratory control ratio; ROS, Reactive oxygen species; RRC, Reserve respiratory capacity; SIRT, Sirtuin; SOD2, Superoxide dismutase‐2; TBA, Thiobarbituric acid; TFAM, Mitochondrial transcription factor A; UCP3, Mitochondrial uncoupling protein 3.

In addition to increasing ROS production, doxorubicin can also suppress cardiac antioxidant defence system. This has been postulated to be partially mediated by alteration in the sirtuins family proteins particularly SIRT1 and SIRT3.^18^ Suppression of sirtuins expression inhibited various endogenous antioxidant enzyme.^18^ Doxorubicin reduced endogenous antioxidant enzyme activity including that of haem oxygenase 1 (HO‐1), superoxide dismutase (SOD) and glutathione peroxidase (GPx), the levels being measured in both in vitro and in vivo models.^18^, ^47^, ^49^, ^57^, ^70^, ^71^, ^72^, ^73^ Transcription and translation of mitochondrial antioxidant enzymes required the activation of nuclear respiratory factor‐1 and 2 (NRF1, NRF2).^20^ Several studies indicated that doxorubicin decreased NRF1, nuclear NRF2, TFAM and HO1 expression.^42^, ^49^, ^52^ These findings suggested that doxorubicin altered the cardiac antioxidant defence system and could potentially disrupt mitochondrial biogenesis. Taken together, these underlies the importance of mitochondria as the mediator of doxorubicin‐induced cardiotoxicity. A summary of reports on cardiac oxidative stress due to doxorubicin is shown in Table 2.

**Figure 1:**
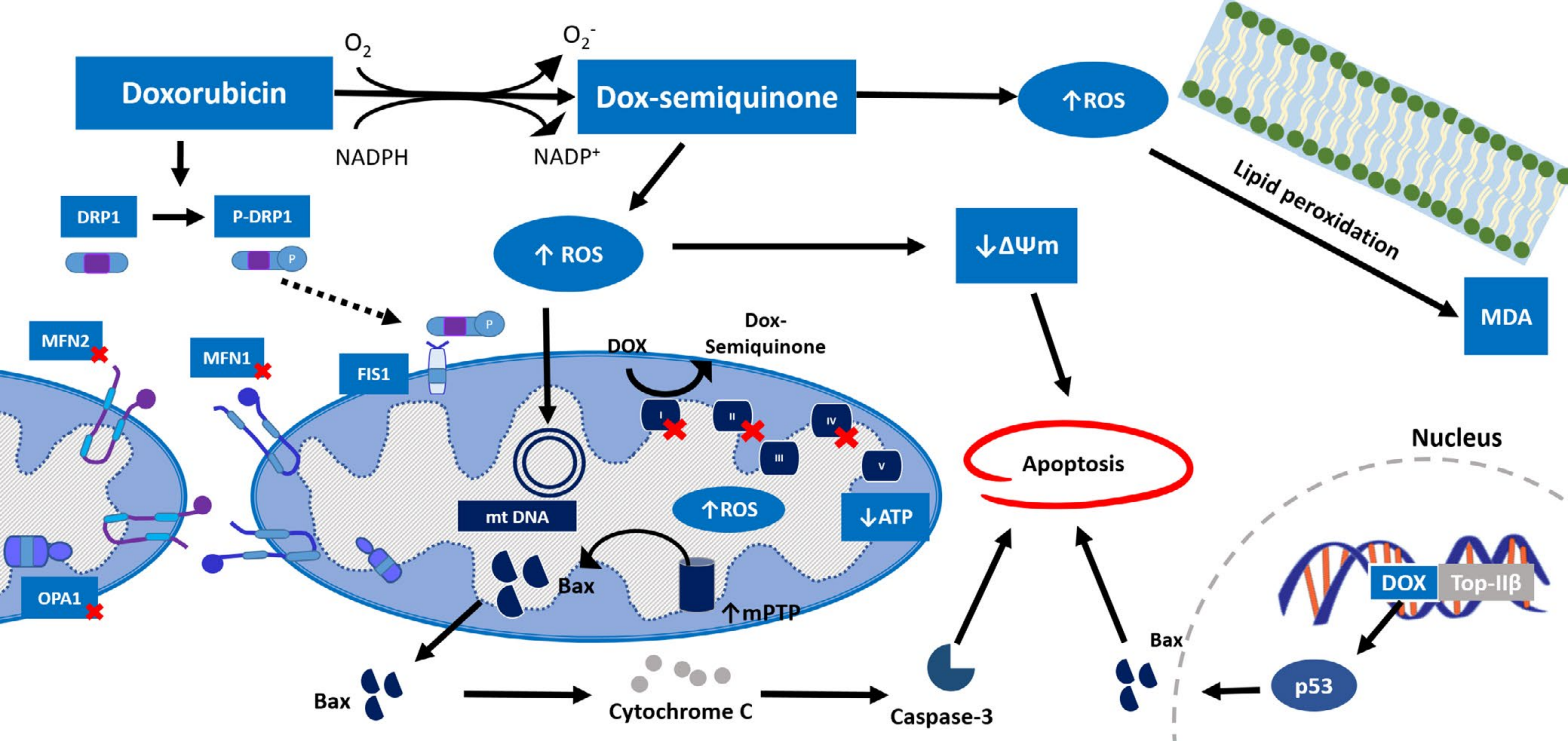
The effects of doxorubicin on cardiac mitochondrial dynamics and mitochondrial function. Doxorubicin inhibits mitochondrial fusion proteins (MFN1, MFN2 and OPA1) and promotes mitochondrial fission by increasing DRP1 phosphorylation. Doxorubicin undergoes redox cycling and generation of ROS. ROS, in turn, induces lipid peroxidation at cellular membrane and targets subcellular organelle causing mitochondrial DNA damage and decreases mitochondrial transmembrane potential. Doxorubicin inhibits electron transport chain proteins subunit I, II, IV and induces mPTP opening which initiates apoptotic signalling pathway. In addition, doxorubicin binds to topoisomerase IIβ in the nucleus causing DNA double‐stranded breaks and induces apoptosis. Dox, Doxorubicin; MDA, Malondialdehyde; mPTP, mitochondrial permeability transition pore; mtDNA, Mitochondrial DNA; ROS, Reactive oxygen species; ΔΨm, Mitochondrial transmembrane potential

Although oxidative stress is the most notable of mechanisms involved in doxorubicin‐induced cardiotoxicity, there is accumulating evidence to suggest that doxorubicin can induce apoptosis through mechanisms that are independent of the ROS production. Doxorubicin has been shown to directly interfere with the nucleus of cardiomyocytes causing DNA damage and secondary activation of p53, thus promoting transcription of pro‐apoptotic proteins and the release of cytochrome c which induced apoptosis.^51^ The effects of doxorubicin on cardiac mitochondrial function and apoptosis are shown in Figure 1. Moreover, alteration in autophagy and mitophagy is another proposed mechanisms in doxorubicin‐induced cardiotoxicity. Mitophagy is the cellular process for removal of damaged mitochondria. Recent in vitro study demonstrated that in human adult ventricular cardiomyocyte cell (AC16) treated with doxorubicin (250 nmol/L/24 h), the PINK1 and Parkin protein expressions were increased with subsequently resulted in mitochondrial biogenesis proteins (PGC‐1α, NRF‐1 and TFAM) suppression and mitochondrial damage.^74^ Consistently, an in vivo study in rats treated with sub‐chronic doxorubicin (2 mg/kg/wk/ip/7 wk) showed a significant increase in PINK1.^32^ These findings suggested that doxorubicin‐induced mitophagy and contributed to mitochondrial dysfunction. However, there is evidence to suggest that doxorubicin inhibited mitophagy.^75^, ^76^ An in vivo study in rats received doxorubicin (4 mg/kg/dose/ip/every 5 d/3 doses) indicated that doxorubicin suppressed Parkin‐mediated mitophagy. Inhibition of mitophagy resulted in accumulation of damaged mitochondria, mitochondrial dysfunction and impairment in heart function.^76^ With regard to the role of autophagy, the autophagic proteins were affected by doxorubicin.^32^, ^77^, ^78^ Doxorubicin altered LC3‐II and increased p62 level.^32^, ^77^, ^78^ There is an emerging consensus that doxorubicin blocked the lysosomal degradation process and increased accumulation of autophagosome and autolysosome; thus, the autophagic process cannot be completed.^79^, ^80^ These findings suggested that doxorubicin dysregulated autophagy and inhibited autophagic clearance which accompanied by ROS production, myocardial dysfunction and apoptosis. Reports on these findings are summarized in Table 2.

## 4 THE ROLE OF AMPK ON DOXORUBICIN‐INDUCED CARDIOTOXICITY

5’ Adenosine monophosphate‐activated protein kinase (AMPK) is a key energy sensor and is activated in response to cellular energy depletion.^81^ Phosphorylation of both AMPK and its downstream regulator acetyl‐CoA carboxylase (ACC) is observed after ATP depletion or an increase in the AMP/ATP ratio.^82^ From in vitro studies, NRCMs treated with doxorubicin (1 µmol/L) showed an increase in the phosphorylation of AMPK (p‐AMPK) at 24 hours resulting in activation of the apoptotic pathway and cell death.^54^, ^56^ However, another in vitro study using the same model and dosage of doxorubicin showed that p‐AMPK measured at 2 hours after doxorubicin treatment was increased.^59^ This result suggested that p‐AMPK was increased in response to acute stress. Nevertheless, another model using H9c2 cell with doxorubicin 10 nmol/L found that p‐AMPK measured at 72 hours afterdoxorubicin therapy was unchanged.^83^ In vivo reports also indicated the same results that p‐AMPK level could possibly depend on the doxorubicin dosage and exposure time.^54^, ^56^, ^59^ Targeting AMPK signalling could be another molecular pathway to mitigate in addressing doxorubicin cardiotoxicity. A summary of these reports is shown in Table 2.

## 5 THE ROLE OF IRON SIGNALLING ON DOXORUBICIN‐INDUCED CARDIOTOXICITY

Iron signalling plays an important role in the mechanism of doxorubicin‐induced cardiotoxicity. Specifically, doxorubicin is able to chelate free iron forming doxorubicin‐iron complexes which promotes oxidative stress.^84^ In addition to the ROS pathway, evidence suggests that doxorubicin cardiotoxicity is also mediated through the oxidative‐independent mechanism which is considered to be a key process.^85^ By interfering with iron regulatory proteins (IRPs), doxorubicin treatment could result in the accumulation of free iron within the cardiomyocytes. This occurs from doxorubicinol, a product of doxorubicin metabolism that removes the Fe‐S cluster from the IRP‐,1 which is then converted into apo‐IRP1 which allows IRP1 binding to the iron‐responsive element (IRE).^86^ The apo‐IRP1 could bind to IRE at the mRNA of several iron signalling proteins including ferritin and transferrin receptor1. This complex process contributes to the inhibition of ferritin synthesis and activation of transferrin receptor expression, thus increasing iron overload. Accumulation of free iron enhances the cardiotoxic effects of doxorubicin. Optimal IRE/IRP interaction is an important factor for achieving balance in iron homeostasis. In addition, doxorubicin could directly interfere with IRE mRNA of ferritin heavy chains (FHC) and suppress ferritin expression.^87^ However, several studies indicated that FHC gene and protein expression are increased following doxorubicin exposure.^43^, ^71^, ^88^ This finding is considered to be a defensive mechanism against cytotoxicity of ROS production. Moreover, a recent in vitro study demonstrated that doxorubicin induced the accumulation of free iron, and this was aggravated by haem degradation process.^89^ Accumulation of iron within mitochondria caused lipid peroxidation on its membrane which triggered a specific type of programmed cell death called ‘ferroptosis’.^89^, ^90^ The ferroptosis is a distinct form of regulated cell death which is an iron‐dependent process. Blocking of ferroptosis showed a reduction of mortality in mice treated with doxorubicin (20 mg/kg).^89^ These reports indicated that doxorubicin can induce cell death through ferroptosis apart from the notable apoptosis. Due to this evidence, prevention of cardiac iron overload by targeting the iron signalling pathway could be an effective strategy in the amelioration of doxorubicin‐induced cardiotoxicity. Moreover, inhibition of ferroptosis could be a potential intervention to prevent doxorubicin‐induced cardiotoxicity. Future studies are needed to elucidate this hypothesis.

## 6 EFFECTS OF PHARMACOLOGICAL AND NON‐PHARMACOLOGICAL INTERVENTIONS ON MITOCHONDRIAL DYNAMICS IN DOXORUBICIN‐INDUCED CARDIOTOXICITY: EVIDENCE FROM IN VITRO AND IN VIVO REPORTS

Since doxorubicin has been shown to alter the level of mitochondrial fusion and fission proteins,^14^, ^30^ targeting the mitochondrial dynamic GTPase proteins could be promising in the prevention of doxorubicin cardiotoxicity. Currently, there is a limited number of studies which have investigated the roles of pharmacological interventions concerning doxorubicin‐induced cardiotoxicity on mitochondrial dynamics in the heart.

Sacubitril/Valsartan (LCZ696) is an angiotensin receptor neprilysin inhibitor. It is a standard treatment in patients with heart failure with reduced ejection fraction (HFrEF). Pretreated H9c2 cell with LCZ696 20 µmol/L for 30 minutes before given doxorubicin (5 µmol/L/24 h) demonstrated that LCZ696 inhibited mitochondrial fission and apoptosis by decreasing DRP1 phosphorylation.^31^ Overexpression of DRP1 abolished the protective effect of LCZ696. These findings indicated that the beneficial effect of LCZ696 is associated with the inhibition of mitochondrial fission. Since LCZ696 inhibited mitochondrial fission and apoptosis, it is possible that LCZ696 might promote mitochondrial fusion regulators (MFN1 MFN2 or OPA1 expression) in response to doxorubicin toxicity. However, this hypothesis needs to be validated in future studies. Another agent targeting DRP1 phosphorylation is polyphenolic ellagic acid (EA). EA is a natural antioxidant compound found in numerous fruits and vegetables. Co‐treatment of EA 10 µmol/L with doxorubicin 10 µmol/L for 18 hours showed that EA inhibited mitochondrial fission and fragmentation by the suppression of BNIP3.^29^ This information provides new insight into the modulation of DRP1 signalling and the inhibition of mitochondrial fission as a potential therapeutic option for alleviating doxorubicin‐induced cardiotoxicity. These reports are summarized in Table 3.

**Table 3:** Effects of pharmacological/non‐pharmacological interventions on mitochondrial dynamics against doxorubicin‐induced cardiotoxicity: reports from in vitro and in vivo studies

Study model	Methods (Drug/Dose/Route/Duration)	Major Findings			Interpretation	Ref
		Mitochondrial fusion	Mitochondrial fission	ROS/Apoptosis		
**in vitro reports**						
H9c2 cell	• Pretreated with LCZ696/20 µmol/L/30 min + Dox/5 µmol/L/24 h • Pretreated with LCZ696/20 µmol/L/30 min + *Drp1*‐expression lentivirus + Dox/5 µmol/L/24 h		↓pSer616DRP1/DRP1 ↔ pSer616DRP1/DRP1	↓Annexin V ↓Cleaved caspase3 ↔Cleaved caspase3	LCZ696 attenuated Dox‐induced apoptosis by decreasing p‐DRP1 and mitochondrial fission	31
Postnatal rat cardiomyocyte	• Dox/10 µmol/L/18 h + **Ellagic** acid/10 µmol/L/18 h		↓pSer616 DRP1 ↓Mitochondrial fragmentation		Ellagic acid suppressed Dox‐induced mitochondrial fission by decreasing p‐DRP1	29
**in vivo reports**						
8‐week‐old male Balb/c mice	• Dox/15 mg/kg/ip/3 times/wk/2 wk + **LCZ696**/60 mg/kg/d/4 wk (Started 1 d after Dox)		↓pSer616DRP1/DRP1	↓Cleaved caspase3	LCZ696 attenuated Dox‐induced apoptosis by decreasing p‐DRP1 and mitochondrial fission	31
Male C57BL/6 mice	• Dox/10 mg/kg/ip/single dose + **Cyclosporin A**/1 mg/kg/ip/alternate d (Follow up at 1.5 wk)	↓*Mfn2* ↓*Opa1* (RNA level)	↓*Mtfp1* ↔*Drp1* ↓Mitochondrial fragmentation		Cyclosporin A prevented mitochondrial fragmentation and alterations in mitochondrial fusion balance	13
6‐week‐old male Sprague Dawley rats	• Dox/2 mg/kg/wk/ip/7 wk + **Free wheel activity** unlimited access 24 h/d (Start 5 wk before Dox) • Dox/2 mg/kg/wk/ip/7 wk + **Treadmill training **5 d/wk/12 wk (Start 5 wk before Dox)	↑MFN1 ↑MFN2 ↑OPA1 ↑MFN1 ↑MFN2 ↑OPA1	↔DRP1 ↔DRP1		Both chronic exercise models attenuated the alteration in mitochondrial dynamics	32
Female C57BL6 mice	Dox/8 mg/kg/wk/ip/4 wk + **Treadmill training** 5 d/wk/8 wk • Dox/8 mg/kg/wk/ip/4 wk + **RESV**/320 mg/kg/d	↑MFN1 ↔MFN2 ↑MFN1 ↑MFN2			Both aerobic exercise training and RESV increased the expression of mitofusin proteins	91

Abbreviations: Dox, Doxorubicin; DRP1, Dynamin‐related protein1; LCZ696, Sacubitril/valsartan; MFN1, Mitofusin1; MFN2; Mitofusin2; OPA1, Optic atrophy1; pSer616, Phosphorylation serine616; RESV, Resveratrol; ROS, Reactive oxygen species.

Doxorubicin inhibits mitochondrial fusion and promotes mitochondrial fragmentation which is accompanied by an increased susceptibility to mPTP opening and ROS generation. Co‐treatment of cyclosporin A, an mPTP inhibitor, with a dose of 1 mg/kg/alternate day in mice treated with doxorubicin (10 mg/kg) indicated that cyclosporine A could normalize the mitochondrial fusion gene *Mfn2* and *Opa1*, thus maintaining the mitochondrial fusion balance and preserving mitochondrial ultrastructural changes.^13^

**Figure 2:**
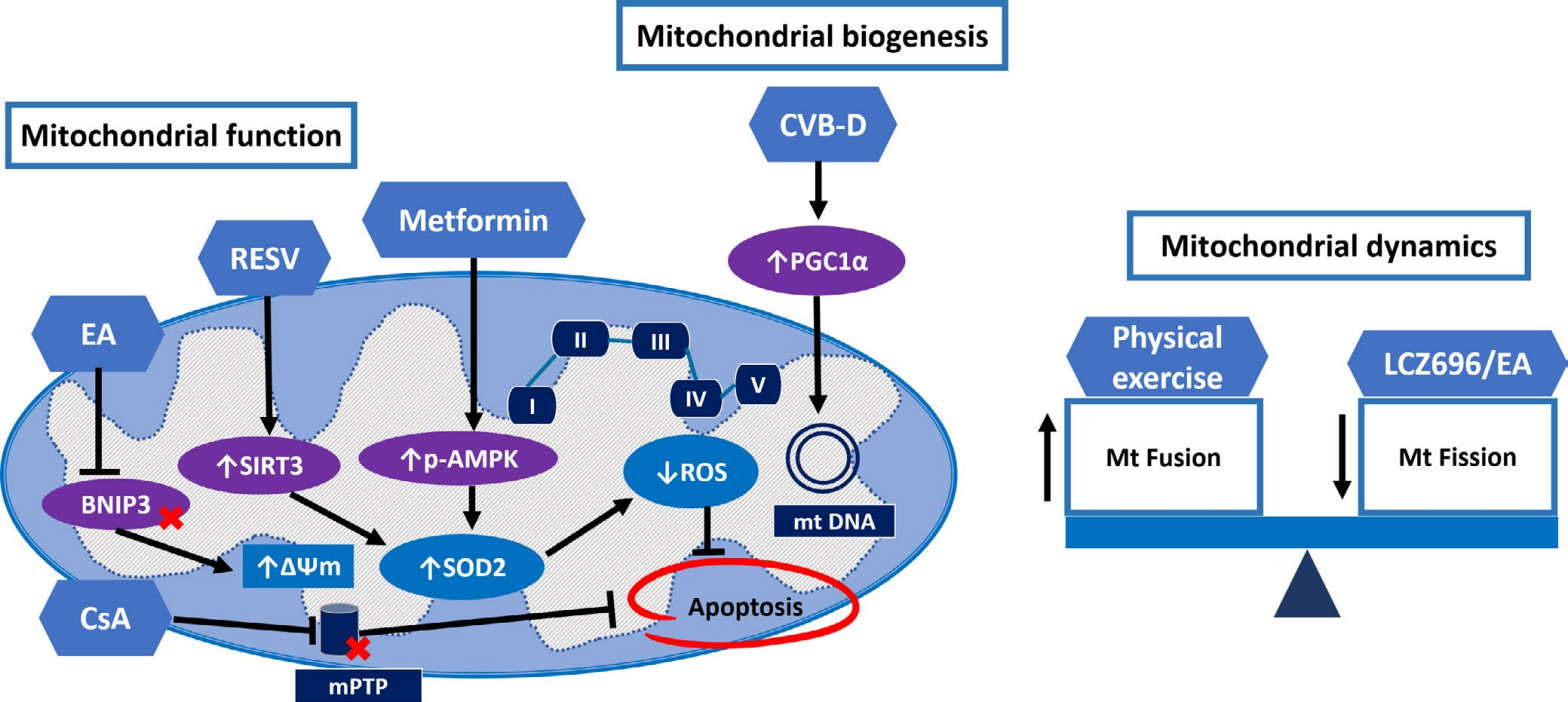
The effects of pharmacological and non‐pharmacological interventions on mitochondrial biogenesis, mitochondrial dynamics and mitochondrial function. Various pharmacological interventions have been shown to attenuate apoptosis by promoting mitochondrial function. The CVB‐D promotes mitochondrial biogenesis by preservation of PGC1α and mitochondrial DNA copy number. Balancing the mitochondrial dynamics by increasing mitochondrial fusion proteins and inhibiting mitochondrial fission process also attenuates ROS production and apoptosis. BNIP3, BCL2/adenovirus E1B 19 kD protein‐interacting protein 3; CsA, Cyclosporin A; CVB‐D, Cycloverobuxine‐D; EA, Ellagic acid; LCZ696, Sacubitril/valsartan; Mt, Mitochondria; mPTP, mitochondrial permeability transition pore; mtDNA, Mitochondrial DNA; RESV, Resveratrol; ROS, Reactive oxygen species; SIRT3, Sirtuin3; SOD2, Superoxide dismutase‐2; ΔΨm, Mitochondrial transmembrane potential

Physical exercise is a non‐pharmacological intervention used in the strategy to reduce cardiac toxicity from doxorubicin. However, the mechanisms responsible for the beneficial effects of exercise are not well characterized. Studies in rats showed that treadmill training and freewheel exercise preconditioning prior to doxorubicin treatment increased the levels of mitochondrial fusion proteins, MFN1, MFN2 and OPA1.^32^, ^91^ It is suggested that the beneficial effects of physical exercise are at least through the regulation of mitochondrial dynamics. The potential therapeutic targets of doxorubicin on mitochondrial dynamics are demonstrated in Figure 2.

Currently, there are limited clinical studies that target mitochondrial dynamics modulation as an intervention in doxorubicin‐induced cardiotoxicity. Future studies are needed to investigate this target and explore whether these interventions can provide cardioprotection in this model.

## 7 EFFECTS OF PHARMACOLOGICAL AND NON‐PHARMACOLOGICAL INTERVENTIONS ON CARDIAC MITOCHONDRIAL FUNCTION IN DOXORUBICIN‐INDUCED CARDIOTOXICITY: EVIDENCE FROM IN VITRO AND IN VIVO STUDIES

The oxidative stress hypothesis is the most widely accepted mechanism for the cause of doxorubicin‐induced cardiotoxicity. Several antioxidant agents have been studied in both in vitro and animal models.^35^, ^57^, ^60^, ^92^ Antioxidants showed cardioprotective effects through reduced ROS generation and decreased apoptosis. However, these effects did not translate into a beneficial outcome in clinical study.^93^, ^94^ Therefore, choosing other interventions that directly promote mitochondrial function could be a promising strategy. Pretreatment or co‐treatment with cyclosporin A, an mPTP inhibitor, has shown beneficial effects by improving mitochondrial respiration and cardiac contractility in both in vitro and in vivo studies.^13^, ^50^ Consistent with the use of ellagic acid (EA), a natural antioxidant which suppressed BNIP3 and promoted mitochondrial function by inhibiting mPTP opening, increasing mitochondria transmembrane potential and reducing cell death in postnatal rat cardiomyocyte.^29^ Furthermore, resveratrol (RESV), a polyphenol found in grapes and berries, exerts cardioprotective effects by promoting SIRT3 expression. SIRT3 is involved in the deacetylation of several mitochondrial proteins and increased SIRT3 expression by RESV attenuated mitochondrial dysfunction and ROS generation in H9c2 cells.^18^ Impairment in mitochondrial biogenesis is considered to be an important process in doxorubicin‐induced cardiotoxicity. PGC‐1α is a key regulator in mitochondrial biogenesis which is inhibited by doxorubicin treatment.^18^, ^41^, ^42^ Cyclovirobuxine D (CVB‐D), a traditional Chinese medicine, has been shown to prevent cardiovascular disease.^95^ Pretreated with CVB‐D 1 mg/kg/d for 4 days before given doxorubicin (15 mg/kg) in mice showed that CVB‐D exerted cardioprotective effects by the preservation of PGC‐1α, NRF1 and mitochondrial DNA copy number.^41^ The potential therapeutic targets of doxorubicin on mitochondrial biogenesis are demonstrated in Figure 2. Regulation in mitochondrial biogenesis could be another effective strategy in the prevention of doxorubicin‐induced cardiotoxicity.

Targeting of iron signalling by the iron chelator, dexrazoxane, has been demonstrated in animal models and translated into clinical trials in cancer patients treated with doxorubicin.^96^ Dexrazoxane significantly reduced the risk of heart failure but had no difference on the survival outcome.^97^ In addition to the iron chelator concept, the cardioprotective effects of dexrazoxane are considered to be involved in the inhibition of anthracyclines binding to Top2β. However, concern about the risk of the development of secondary malignancies ^98^ limits dexrazoxane usage to patients receiving a cumulative dose of doxorubicin of more than 540 mg/m^2^ according to the European Medicine Agency (EMA).

Choosing the interventions that have been widely used in clinical practice could be a favourable way due to the less concern of their adverse reactions. In this regard, beta blocker, statin and metformin have been studied in doxorubicin model. For statin reports, the proposed cardioprotective mechanism of lovastatin is associated with the inhibition of RAC1 signalling with subsequent reduction in apoptosis.^99^, ^100^ A recent in vivo study showed that lovastatin attenuated mitochondrial dysfunction by reducing mitochondrial proliferation.^101^ Atorvastatin and rosuvastatin also have been shown to prevent doxorubicin‐induced cardiotoxicity by reducing oxidative stress and inhibition of apoptosis.^102^, ^103^ In addition to statin, beta blocker has also been investigated in doxorubicin model. For example, carvedilol possesses a distinct cardioprotective properties due to its antioxidant effects and the ability to inhibit lipid peroxidation within myocardial cells.^104^, ^105^ The studies in animal models indicated that co‐administration of carvedilol with doxorubicin prevented the inhibitory effects of doxorubicin on mitochondrial respiration.^106^ Carvedilol also prevented mitochondrial damage and the decrease in mitochondrial calcium loading capacity in rats treated with doxorubicin.^104^, ^106^ These findings suggested that carvedilol could prevent cardiac mitochondrial dysfunction in doxorubicin model.

Several reports from both in vitro and in vivo have demonstrated that metformin had a cardioprotective role in doxorubicin model. The findings from in vitro studies indicated that the adiponectin system and AMPK could both play a role in the preventive effects of metformin (Table 4). It has been shown that inhibition of adiponectin receptor1 (adipoR1) and adiponectin receptor2 (adipoR2) abrogated the protective effects of metformin in HL‐1 cell.^70^ Since adiponectin could activate AMPK, this finding indicated the regulation of AMPK as the mechanism responsible for the attenuation of the doxorubicin‐induced cardiotoxicity. Consistent with another report using H9c2 cell which demonstrated the efficacy of the cardioprotective effects of low‐dose metformin through the increase in p‐AMPK and its downstream regulators.^83^ However, a recent report showed that berberine, a natural alkaloid extracted from a variety of plants, improved mitochondrial function and decreased myocardial apoptosis by inhibiting AMPK phosphorylation in rats and NRCMs treated with doxorubicin.^59^ The inconsistent effects of AMPK activation in the doxorubicin model could be due to differences in dosage and doxorubicin exposure time. Further studies concerning the role of AMPK intervention in attenuating doxorubicin‐induced cardiotoxicity are needed.

**Table 4:** Effects of pharmacological/non‐pharmacological interventions on mitochondrial function against doxorubicin‐induced cardiotoxicity: reports from in vitro and in vivo studies

Study model	Methods (Drug/Dose/Route/Duration)	Major Findings					Interpretation	Ref
		Heart function/Morphology	Oxidative phosphory lation	Autophagy Mitophagy	ROS/poptosis	MMP/mPTP/ Mitochondrial protein		
**In vitro reports**								
Human right atrial trabeculae	• Dox/1 µmol/L/0‐90 min + **Cyclosporin A**/1 µmol/L/10 min before dox	↑Developed force ↑Maximal contraction velocity ↑Maximal relaxation velocity	↓State 2 ↑State 3 ↑RCR			↑MMP ↓mPTP	Cyclosporin A inhibited mPTP opening improved mitochondrial respiration and cell contraction	50
Postnatal rat cardiomyocyte	• Dox/10 µmol/L/18 h + **Ellagic acid**/10 µmol/L/18 h				↓ROS ↓LDH ↓%Dead cells	↑MMP ↓mPTP ↓Mitochondria BNIP3	EA suppressed mitochondrial injury and cell death by abrogating BNIP3 activity	29
H9c2 cell	• Dox/1 µmol/L/24 h + **RESV**/50 µmol/L/24 h ***Sirt3*‐null MEFs** • Dox/1 µmol/L/6 h ***Sirt3*‐null MEFs** • Dox/1 µmol/L/6 h + **RESV**/10 µmol/L/6 h				↑SOD2 ↓ROS ↑↑ROS ↔SOD2 ↑ROS	↑SIRT3 ↔SIRT1 ↓PGC1α acetylation	SIRT3 expression was necessary for RESV to attenuate Dox‐induced ROS production	18
H9c2 cell	• Dox/5 µmol/L/24 h + **Pretreated with LCZ696**/20 µmol/L/30 min • Dox/5 µmol/L/24 h + **Pretreated with LCZ696**/20 µmol/L/30 min + *Drp1*‐expression lentivirus (OE cell)	↑Mt size ↔Mt size			↓AnnexinV ↓Cleaved caspase3 ↔Cleaved caspase3		LCZ696 preserved mitochondrial morphology and decreased apoptosis activity	31
Neonatal Sprague Dawley rat cardiomyocyte	**Pretreated with Berberine/1 µmol**/L**/20 min** • Dox/1 µmol/L/2‐24 h				↓p‐p53 ↓Bax ↑Bcl‐2 ↓TUNEL‐positive	↑MMP ↓p‐AMPKα ↓AMP/ATP ↓p‐ACC	Berberine suppressed Dox‐induced cardiomyocyte apoptosis through the inhibition of AMPK phosphorylation	59
HL‐1 cell	• Dox/5 µmol/L/15 or 24 h + **Metformin**/4 mmol/L/24 h **Transfected with *AdipoR1* or *AdipoR2* siRNA** • Dox/5 µmol/L/15 h + **Metformin**/4 mmol/L/24 h				↓Caspase 3,9 activity ↓TUNEL‐positive cells **↑**Cell viability ↑Catalase activity ↑GPx activity ↑SOD activity ↔Cell viability ↔Catalase activity ↔GPx activity ↔SOD activity		The protective effects of metformin against Dox‐induced cardiotoxicity were considered to be involved in the regulation of the adiponectin system	70
H9c2 cell	• Dox/10 nmol/L/1‐72 h + **Metformin**/0.1 mmol/L/1‐72 h • Dox/10 nmol/L/1‐72 h + **Metformin**/1.0 mmol/L/1‐72 h **Treated with AMPK inhibitor 10 µmol**/L • Dox/10 nmol/L/1‐72 h + **Metformin**/0.1 mmol/L/1‐72 h				↓LDH ↓ROS ↑Cell viability ↔Cell viability ↔LDH ↔ROS ↔Intracellular calcium ↔ Cell viability	↑p‐AMPK (72 h) ↑p‐ACC ↑PKA activity ↑p‐PDGFRβ ↑p‐AMPK (72 h) ↑p‐ACC ↑PKA activity ↓p‐PDGFRβ ↔PKA activity	Low‐dose metformin exerted cardioprotective effects against Dox by regulating AMPK pathway High‐dose metformin reverted the protective effects by suppressing PDGFR expression	83
**In vivo reports**								
Male C57BL/6 mice	• Dox/10 mg/kg/ip/single dose + **Cyclosporin A**/1 mg/kg/ip/alternate day (Follow up at 1.5 wk)	↑LVFS ↔Mt density ↔Mt size ↑Mt elongated	↑RCR			↓mPTP ↔PGC1α ↔Mt DNA	Cyclosporin A inhibited mPTP opening, mitochondrial potential loss and contractile depression	13
8‐week‐old male Balb/c mice	• Dox/15 mg/kg/ip/3 times/wk/2 wk + **LCZ696**/60 mg/kg/d/4 wk (Start 1 d after Dox)	↑LVEF ↓LVEDD ↓LVESD ↔Mt width ↑Mt length ↔Mt length/width	↑Complex I ↔Complex IV		↓Cleaved caspase3 ↓TUNEL staining		LCZ696 improved cardiac function, mitochondrial respiration and decreased apoptosis	31
C57BL mice	**Pretreated with CVB‐D 1 mg/kg/d/4 d** • Dox/15 mg/kg/ip/single dose	↑LVEF ↑FS ↓Myocardial swelling, vacuolization			↓Lipid peroxidation ↓Protein carbonylation ↑GSH/GSSG ↓Cytosolic cytochrome c ↓TUNEL‐positive	↑PGC1α ↑NRF‐1 ↑mtDNA copy number	CVB‐D protected against Dox‐induced cardiomyopathy by suppression of oxidative damage and mitochondrial biogenesis impairment	41
Male Sprague Dawley rats	• Dox/20 mg/kg + **Berberine**/60 mg/kg/dose/4 d	↑LVEDV ↑Stroke volume ↔LVEF			↓p‐p53 ↑Bcl2 ↓Cleaved caspase3 ↓TUNEL‐positive ↑Survival	↓p‐AMPK	Berberine attenuated Dox‐induced apoptosis by increased Bcl2 expression and decreased p53‐AMPK pathway	59
Male Sprague Dawley rats	• Dox/2 mg/kg/wk/sc/7 wk •Dox/2 mg/kg/wk/sc/7 wk + **Carvedilol/**1 mg/kg/wk/ip/7 wk	↑Swollen mitochondria ↓Swollen mitochondria	↓Stage3 ↔Stage4 ↓RCR ↓Complex I ↑Stage3 ↔Stage4 ↑RCR ↑Complex I				Carvedilol prevented the inhibitory effects of Dox on mitochondrial respiration and mitochondrial morphology damage	106
Adult male Wistar Albino rats	• Dox/3 mg/kg/EOD/ip/6 doses + **Metformin**/50 mg/kg/d/oral/11 d • Dox/3 mg/kg/EOD/ip/6 doses + **Metformin**/500 mg/kg/d/oral/11 d	No Myocardial firbe injury ↓Inflammatory infiltration Normal myocardial fibres			↔CK‐MB ↔LDH ↑GSH ↔GSTα ↔HO‐1β ↔CAT ↔NQO1 ↓CK‐MB ↓LDH ↑GSH ↓GSTα ↓HO‐1β ↑CAT ↑NQO1	↔Acetyl‐CoA ↔ATP ↓Acetyl‐CoA ↑ATP	Metformin prevented Dox‐induced cardiotoxicity by inhibiting Dox‐induced oxidative stress and energy starvation	72
Male Wistar rats	• Dox/15 mg/kg/ip/single dose + **Metformin**/250 mg/kg/d/oral/7 d	↓Myocardial degeneration ↓Interstitial inflammation ↓Interstitial haemorrhage			↓LDH ↓CK‐MB ↓MDA ↑SOD ↓COX‐2 ↓Caspase3		Metformin attenuated Dox‐induced cardiotoxicity in rats due to its antioxidant, anti‐inflammatory and anti‐apoptotic properties	107
Male Wistar Albino rats	• Dox/20 mg/kg/ip/divided into 2 doses + **Metformin**/500 mg/kg/d/oral/7 d	↓Myocyte degeneration ↓Interrupted muscle fibre ↓Wide interstitial spaces			↓CK‐MB ↓LDH ↑GSH ↓TBA ↓Caspase3 ↑Bcl2	↓TNF‐α ↔Blood glucose	Metformin exerted protective effects against Dox‐induced cardiotoxicity by inhibition of apoptotic pathway	73
Male Wistar Albino rats	• Dox/4 mg/kg/dose/ip/twice a week/4 doses + **Metformin**/250 mg/kg/d/oral/14 d	↑LVEF ↓LVESD Almost normal histology			↔Catalase ↔SOD ↔GPx ↔Apoptotic cells	↔TNF‐α ↔BNP	Metformin preserved contractile function and attenuated histological damage	53
Sprague Dawley rats	• Dox/3 mg/kg/EOD/ip/6 doses + **Metformin**/250 mg/kg/d/oral/14 d	↑Aortic flow ↑Cardiac output ↔Stroke volume ↑Myocardial thickness		↔Beclin‐1 ↑LC3B‐II ↔p62	↔LDH ↔CK‐MB ↓Trop T ↓MDA	↔p‐AMPK	Administration of metformin with Dox normalized the autophagic activity and conferred cardioprotection	78
6‐week‐old male Sprague Dawley rats	• Dox/2 mg/kg/wk/ip/7 wk + **Free wheel activity** unlimited access 24 h/d (Start 5 wk before Dox) • Dox/2 mg/kg/wk/ip/7 wk + **Treadmill training **5 d/wk/12 wk (Start 5 wk before Dox)			↔Beclin1 ↓Beclin1/Bcl2 ratio ↓LC3‐II ↔p62 ↓PINK1 ↔Parkin ↔Beclin1 ↓Beclin1/Bcl2 ratio ↓LC3‐II ↔p62 ↓PINK1 ↔Parkin	↓Bax/Bcl2 ratio ↓Caspase3,9 ↓Bax/Bcl2 ratio ↓Caspase3,9	↓mPTP ↓mPTP	Both chronic exercise models attenuated apoptotic signalling and alterations in autophagy	32
Female C57BL6 mice	• Dox/8 mg/kg/wk/ip/4 wk + **Treadmill training **5 d/wk/8 wk • Dox/8 mg/kg/wk/ip/4 wk + **RESV**/320 mg/kg/d	↔LVEF ↑LVEF	↑Complex I ↑Complex II ↔Complex IV ↑Complex I ↑Complex II ↑Complex IV		↓4‐HNE ↑MnSOD ↓4‐HNE ↔MnSOD		Both aerobic exercise training and RESV reduced oxidative stress, promoted expression of mitochondrial electron transport chain proteins and improved heart function	91

Abbreviations: ACC, Acetyl‐CoA carboxylase; AdipoR, Adiponectin receptor; BNIP3, BCL2/adenovirus E1B 19 kD protein‐interacting protein 3; CAT, Catalase; COX1, Cytochrome c oxidase subunit1; CVB‐D, Cyclovirobuxine; Dox, Doxorubicin; GPx, Glutathione peroxidase; GSH, Reduced glutathione; GSSG, Oxidized glutathione; GSTα, Glutathione S‐transferase‐α; HO‐1β, Haem oxygenase‐1β; LCZ696, Sacubitril/valsartan; LVEDD, Left ventricular end‐diastolic dimension; LVEDV, Left ventricular end‐diastolic volume; LVEF, Left ventricular ejection fraction; LVESD, Left ventricular end‐systolic dimension; LVFS, Left ventricular fractional shortening; MDA, Malondialdehyde; MMP, Mitochondrial membrane potential; MnSOD, Manganese superoxide dismutase; mPTP, Mitochondrial permeability transition pore; Mt, Mitochondria; NQO1, NAD(P)H:quinone oxidoreductase 1; NRF, Nuclear respiratory factor; OCR, Oxygen consumption rate; P, Phosphorylation; PDGFRβ, Platelet‐derived growth factor receptor β; PKA, Protein kinase A; RCR, Respiratory control ratio; RESV, Resveratrol; ROS, Reactive oxygen species; SIRT, Sirtuin; SOD2, Superoxide dismutase‐2; TBA, Thiobarbituric acid.

Evidence from in vivo studies also supports the protective effect of metformin in doxorubicin models (Table 4). Co‐treatment with metformin in rats demonstrated that metformin exerted cardioprotective effects by increasing cardiac antioxidant enzyme level including reduced glutathione (GSH) and SOD.^72^, ^107^ The potential therapeutic targets of doxorubicin on mitochondrial function are demonstrated in Figure 2. Moreover, co‐treatment with metformin in Wistar rats showed that metformin attenuated apoptosis afterdoxorubicin therapy.^73^, ^107^ These effects contributed to the preservation of mitochondrial morphology and attenuated myocardial damage.^72^, ^73^, ^107^ Another proposed mechanism for the cardioprotective effects of metformin is its role in the regulation of autophagy. Doxorubicin impaired autophagy by altering the process of autophagosome formation and inhibition of autophagic clearance.^32^, ^77^, ^78^ Co‐treatment with metformin normalized the expression of autophagic enzymes and mitigated the cardiotoxic effects of doxorubicin.^78^ A summary of the reports of all of these findings is shown in Table 4

With regard to non‐pharmacological intervention, various physical exercises have been shown to be the effective interventions against doxorubicin‐induced cardiotoxicity. Treadmill training in mice treated with doxorubicin showed a reduction in oxidative stress, increased expression of mitochondrial electron transport chain proteins and an improved heart function.^91^ The beneficial effects of swim training was involved in an increase in heat shock proteins of the 60 kD family (HSP60).^108^ Furthermore, both treadmill training (TM) and free wheel activity (FW) could normalize the increase in the autophagic initiation protein, beclin‐1/bcl2 ratio and reduced apoptosis.^32^ This finding suggested that lower intensity and longer duration (FW) may be as protective against doxorubicin toxicity as higher intensity and shorter duration exercise (TM). These data strengthen the role of physical exercise in attenuating doxorubicin‐induced cardiotoxicity apart from the regulation in mitochondrial dynamics. The comprehensive summary of these findings is shown in Table 4.

Various pharmacological and non‐pharmacological interventions have been studied in both in vitro and animal model as we have discussed. Whether these interventions contribute to beneficial outcome in clinical trials are inconsistent and controversial.^109^, ^110^, ^111^ Most of the clinical trials evaluated only heart function and did not demonstrate the possible involved cardioprotective mechanism.^109^, ^110^, ^111^ In addition, there is lack of clinical study which investigate the effects of the intervention drugs on mitochondrial dynamics and function. Further clinical studies in this field are needed to improve outcome in these patients.

## 8 CONCLUSION

The mechanisms involved in doxorubicin‐induced cardiotoxicity are complex. Oxidative stress, mitochondrial dysfunction and apoptosis play an important role in the development of cardiomyopathy. The balance of mitochondrial dynamics and normal mitochondrial function are disrupted by doxorubicin which results in myocardial damage. Successful identification of interventions that could attenuate doxorubicin‐induced cardiotoxicity would be of tremendous clinical benefit for cancer patients treated with doxorubicin.

## CONFLICT OF INTEREST

None.

## AUTHOR CONTRIBUTIONS

NO performed the literature search, drafted the manuscript, made the figure and tables; AP, SCC, NC designed the concept and revised the manuscript; NC revised the manuscript and provided final approval of the version to publish.
